# Blood concentrations of α-Klotho and FGF-23 exhibit no correlation with bone mineral density in elderly individuals

**DOI:** 10.31744/einstein_journal/2024AO0412

**Published:** 2024-09-09

**Authors:** Karina Moura Sawada, Niele Silva de Moraes, Lara Miguel Quirino Araújo, Fernanda Martins Gazoni, Marise Lazaretti-Castro, Maysa Seabra Cendoroglo, John P. Bilezikian, Maria Stella Figueiredo, Fania Cristina dos Santos

**Affiliations:** 1 Universidade Federal de São Paulo Escola Paulista de Medicina Department of Medicine São Paulo SP Brazil Discipline of Geriatrics, Department of Medicine, Escola Paulista de Medicina, Universidade Federal de São Paulo, São Paulo, SP, Brazil.; 2 Universidade Federal de São Paulo Escola Paulista de Medicina Department of Medicine São Paulo SP Brazil Discipline of Endocrinology, Department of Medicine, Escola Paulista de Medicina, Universidade Federal de São Paulo, São Paulo, SP, Brazil.; 3 Columbia University College of Physicians and Surgeons Department of Medicine New York New York US Division of Endocrinology, Department of Medicine, College of Physicians and Surgeons, Columbia University, New York, New York.; 4 Universidade Federal de São Paulo Escola Paulista de Medicina Department of Clinical and Experimental Oncology São Paulo SP Brazil Discipline of Hematology and Hemotherapy, Department of Clinical and Experimental Oncology, Escola Paulista de Medicina, Universidade Federal de São Paulo, São Paulo, SP, Brazil.

**Keywords:** Biomarkers, Klotho protein, FGF-23 protein, Human, Bone density, Aged, Longevity

## Abstract

**Objective:**

To investigating the relationship between α-Klotho and FGF-23 with bone biochemical markers and bone density findings in extremely aged individuals.

**Methods:**

A total of 55 individuals with a mean age of 85.6 years were subjected to clinical, biochemical, and bone mineral density analyses and the enzyme-linked immunosorbent assay-based detection of α-Klotho and FGF-23. The mean, standard deviation, median, and interquartile ranges of the sample values were determined, and Spearman's test for association assessments was used for statistical analysis.

**Results:**

The study participants expressed median FGF-23 and α-Klotho levels of 69.81 RU/mL (51.43 RU/mL) and 733.43 pg/mL (360.83 pg/mL), respectively. The majority of the participants possessed osteopenia (54.5%) and a vitamin D deficiency (57%). The 25-hydroxyvitamin D concentrations ranged between 7.1 and 47.5ng/mL, with a median of 18.1ng/mL.

**Conclusion:**

No substantial associations were discovered between α-Klotho and FGF-23 levels and bone density in the study participants.

## In Brief

Sawada et al. investigated the relationship between α-Klotho and FGF-23 with bone biochemical markers and bone density findings in elderly individuals. The authors did not observe any notable associations between Klotho and FGF-23 levels or alterations in bone mineral density. Despite this, the present study employed solely extremely aged participants, which distinguishes it from the surveyed populations of other studies.

## Highlights

Klotho controls cellular pathways and is a key factor in senescence and longevity.
FGF-23 regulates phosphorus and vitamin D metabolism in a Klotho-dependent manner.
Bone formation in Klotho-deficient mice decreased similar to that in aged humans.
Renal function and lumbar spine bone mineral density were related to Klotho and FGF-23, respectively.

## Graphical abstract

**Figure:**
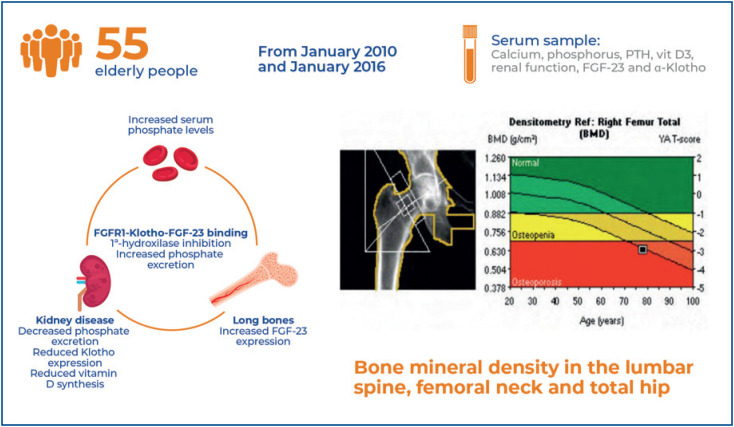

## INTRODUCTION

The proportion of elderly individuals worldwide is experiencing a surge greater than any other age group, and the upper age limit (80 years and older) is rapidly escalating.^(1)^ This aging population makes it imperative to devise strategies aimed at increasing the number of years lived as "healthy" individuals.

Aging and longevity are multifactorial events. Researchers have observed that approximately 25% of the overall variation in life expectancy can be attributed to genetic factors, which in turn are strikingly relevant to extreme longevity.^(2)^

Despite constant advancements in research on aging, it is challenging to ascertain the molecular biomarkers of biological age or the rate of aging.^(3)^The *Klotho* gene has been identified as a key factor in senescence and longevity owing to its role in different cellular pathways.^(4)^ The *Klotho* gene was discovered in 1997 and originally identified as a putative age suppressor. Subsequent research has revealed that defective *Klotho*expression in mice triggers a syndrome resembling premature aging in humans (cognitive impairment, atherosclerosis, sarcopenia, and osteoporosis); conversely, mice overexpressing *Klotho* were reported to exhibit extended, healthy lifespans.^(4-6)^

The Klotho protein family is composed of three distinct single-pass transmembrane proteins: α-Klotho, β-Klotho and Klph (protein related to Klotho-lactase-flotizine hydrolase or γ-Klotho).^(4)^ The name "Klotho" is conventionally used to refer to α-Klotho, the designation for the original *klotho* gene and its product. The Klotho protein is predominantly expressed in the distal renal tubules, choroid plexus, and pituitary gland and exists in two forms: membrane-bound and secreted Klotho. Klotho is a co-receptor for the fibroblast growth factor 23 (FGF-23) in the membrane and assumes essential roles in phosphate regulation and vitamin D synthesis in the kidneys. Soluble Klotho is a humoral factor with pleotropic activities, including regulation of nitric oxide production in the endothelium, modulation of ion channels and transporters, and inhibition of intracellular insulin and insulin-like growth factor-1 signaling.^(5-7)^

FGF-23 is a hormone that regulates phosphorus and vitamin D metabolism in a Klotho-dependent manner. It is secreted by osteocytes and functions efficiently through interaction with the "Klotho-FGF-23 receptor" complex in the membrane of the proximal renal tubule cells, inducing urinary phosphate excretion. Furthermore, binding between Klotho and FGF-23 creates a negative feedback loop, which contains an enzyme that converts 25-hydroxyvitamin D to its active form (25-dihydroxyvitamin D).^(8)^

The Klotho-FGF-23 complex forms a crucial endocrine axis for the regulation of mineral metabolism and assumes a key role in the pathophysiology of age-related disorders, including diabetes, cancer, arteriosclerosis, and chronic kidney disease.^(6,7)^ Several studies have evidenced that the concentration of FGF-23 increases early in chronic kidney disease (CKD) and is considered a biomarker that integrates the reduction in glomerular filtration rate.^(6-9)^

Accumulating evidence has substantiated the involvement of genetic factors in decreasing in bone mineral density. Osteoporosis is a systemic bone disease characterized by reduced bone density and altered skeletal architecture. This results in a heightened susceptibility to bone fracture, morbidity, and mortality. An investigation on Klotho-deficient mice demonstrated suppressed bone formation similar to that observed during human aging.^(10)^ Therefore, efforts to identify new biomarkers could improve the assessment of fracture risk and other complications associated with bone metabolism disorders in elderly individuals.

## OBJECTIVE

To investigate the relationship between α-Klotho and FGF-23 with bone biochemical markers and bone density findings in extremely aged individuals.

## METHODS

A cross-sectional study of the "Longevos Project," of the discipline of geriatrics and gerontology, was conducted at *Escola Paulista de Medicina, Universidade Federal de São Paulo*. This project refers to a prospective cohort that commenced in 2010 and is still in progress, with elderly participants aged ≥80 years of both sexes living independently in the community. The exclusion criteria were as follows: diagnosis of dementia, severe acute illness, or decompensated chronic illness; undergoing dialysis, chemotherapy, or radiotherapy; current neoplasm, except non-melanoma skin neoplasm; hospitalization within 3 months prior to the experiment; history of cerebrovascular accident or heart attack with significant limitations; and visual or auditory *deficits*, which made it impossible to answer the questionnaires.

This study was approved by the Research Ethics Committee of the *Universidade Federal de São Paulo* (CAAE: 09962213.0.0000.5505; #202,943). All the participants provided written, informed consent. Fifty-five elderly individuals were evaluated between January 2010 and January 2016.

### Data collection and assessment of clinical parameters

The study participants answered a structured questionnaire requesting data pertaining to sociodemographic background, current and previous diseases, smoking history, and medication prescriptions.

### Laboratory tests

Serum samples were collected from all participants following an 8-hours fast. Serum levels of calcium, phosphorus, intact parathyroid hormone, and 25-hydroxyvitamin D3 were evaluated along with renal function (the CKD-EPI creatinine-cystatin C formula was used to estimate glomerular filtration rate). Serum specimens were examined to quantitate FGF-23 levels using enzyme-linked immunosorbent assay (ELISA; Immutopics; Immutopics International, San Clemente, CA, USA), which detects two epitopes at the C-terminus of FGF-23 with a sensitivity of 1.5 relative units per mL (RU/mL); the inter- and intra-assay coefficients of variation were established at <5%. Expression levels of α-Klotho were measured by analyzing plasma samples using solid phase sandwich ELISA kits (DuoSet ELISA, Cat # DY5334-05 and DY2604-05; R&D Systems, Minneapolis, MN, USA).

Plasma samples obtained previously were thawed, and 100µL of solution containing monoclonal antibody against human Klotho protein diluted in phosphate-buffered saline (PBS; capture antibody) was added to each well of the DuoSet ELISA plate. The plates were incubated for at least 12 hours at room temperature. Antibodies that failed to adhere to the plates were discarded by inversion, and the plates were washed with 0.05% PBS-Tween. Subsequently, the plates were blocked with 300µL/well of a 1% bovine serum albumin (BSA)-containing solution for at least 1 hour at room temperature. After washing the plates again, 100µL of the sample or standard was incorporated into each well, following which the plates were incubated for at least 2 hours at room temperature and then washed. Antibodies conjugated with biotin and diluted in 0.1% BSA were incubated for 2 hours at room temperature. Next, following another wash, 100µL/well of streptavidin-horseradish peroxidase were added to the plates, which were incubated for 20 minutes at room temperature. Finally, after a last wash, a substrate solution comprising H_2_O_2_ and tetramethylbenzidine in a 1:1 ratio was added to the plates and incubated in the dark. The reaction was terminated using a solution containing sulfuric acid. The labeling intensity was recorded using an ELISA reader at a wavelength of 450nM.

### Densitometric data

Bone mineral density (BMD) was measured via dual-energy X-ray absorptiometry using specimens extracted from the lumbar spine, femoral neck, and total hip (Hologic).

### Statistical analysis

The adequacy of the variables for normal distribution was examined using a histogram, skewness, and the kurtosis method.

Descriptive analyses were performed using the mean and standard deviation for normally distributed variables and the median and interquartile range (IQR for non-normally distributed variables.

Spearman's correlation coefficient test was used to determine the presence or absence of any association between variables. The Spearman correlation coefficient (r_s_) can vary from +1 (positive association) to -1 (negative association). Values of r_s_ exceeding 0.4 indicate a strong correlation between variables; values between 0.2 and 0.4 are considered moderate, and those below 0.2 are deemed weak.

A significance level of 5% was established for all statistical tests, and analyses with a descriptive level (p) lower than 0.05 were considered statistically significant.

## RESULTS

Demographic and biochemical characteristics of the study participants have been presented in table 1. Correlation analysis of all study variables with α-Klotho and FGF-23 have been depicted in tables 2 and 3, respectively.

**Table 1:** Characteristics of the study participants

Demographics		Total mean ± SD or (%)
Age (years)		85.63±4.02
Sex		
	Female	43 (78)
	Male	12 (22)
Race		
	White	33 (65)
	Black	4 (8)
	Brown	10 (20)
	Others	4 (8)
**Biochemicals**		**Median (IQR) or mean ± SD**
	Creatinine (mg/dL)	0.885 (0.29)
	CKD-EPI (mL/min/1.73m^2^)	53.79±14.27
	25(OH)VITD3 (ng/mL)	18.1 (8.6)
	PTH (pg/mL)	62.32±26.5
	Calcium (mg/dL)	9.5±0.45
	Phosphorus (mg/dL)	3.4 (0.5)
	FGF-23 (RU/mL)	69.81 (51.43)
	α-Klotho (pg/mL)	733.43 (360.83)
**BMD/T-score**		**Median (IQR) or mean SD**
>Lumbar spine		
	BMD	0.892 (0.187)
	T-score	-1.6 (1.4)
Femoral neck		
	BMD	0.68 ± 0.12
	T-score	-1.9 (1.1)
Total hip		
	BMD	0.7485 (0.1615)
	T-score	-1.6 (1.1)

SD: standard deviation; IQR: interquartile range; BMD: bone mineral density.

**Table 2:** Correlation of α-Klotho with study variables

	rs (n)	p value
Age (years)	- 0.0836 (55)	0.5438
Creatinine (mg/dL)	-0.1697 (54)	0.2200
CKD-EPI (mL/min/1.73m^2^)	0.2399 (54)	0.0806
25(OH)VitD (ng/mL)	0.0138 (45)	0.9285
PTH (pg/mL)	0.0132 (45)	0.9315
Calcium (mg/dL)	0.0458 (45)	0.7649
Phosphorus (mg/dL)	-0.0864 (45)	0.5727
BMD lumbar spine	-0.0414 (53)	0.7683
T-score lumbar spine	0.0417 (55)	0.7624
BMD femoral neck	-0.0143 (52)	0.9201
T-score femoral neck	0.0856 (54)	0.5384
BMD total hip	-0.0112 (52)	0.9370
T-score total hip	0.0666 (54)	0.6321
FGF-23 (ng/L)	0.0635 (27)	0.7530

r_s_: Spearman's correlation test.

**Table 3:** Correlation between FGF-23 and study variables

	r_s_ (n)	p value
Age (years)	-2.949 (27)	0.1353
Creatinine (mg/dL)	0.1170 (27)	0.5611
CKD-EPI (mL/min/1.73m^2^)	-0.0336 (27)	0.8679
25(OH)VitD (ng/mL)	0.0528 (27)	0.7936
PTH (pg/mL)	0.1728 (27)	0.3888
Calcium (mg/dL)	-0.1628 (27)	0.4172
Phosphorus (mg/dL)	0.3182 (27)	0.1058
BMD lumbar spine	-0.2315 (26)	0.2553
T-score lumbar spine	-0.1995 (27)	0.3185
BMD femoral neck	0.0684 (26)	0.7399
T-score femoral neck	0.1438 (27)	0.4742
BMD total hip	-0.0112 (26)	0.9370
T-score total hip	0.0619 (27)	0.7639

r_s_: Spearman's correlation test.

The Spearman test results substantiated that TFG and lumbar spine BMD displayed potential moderate associations with Klotho (r=0.2399, p=0.0806) and FGF-23 (r=-0.2315, p=0.2553), respectively; however, the findings were not statistically significant. For the other variables, no plausible associations were discovered with α-Klotho and FGF-23, and none were statistically significant.

## DISCUSSION

The action of Klotho as an anti-aging hormone or a predictive marker of renal dysfunction is unclear.^(11)^ Polymorphisms in the *klotho* gene have already been significantly associated with aging, particularly those related to osteoblasts.^(12,13)^ Acting on TRPV5, a recently identified modulator of bone function, the *klotho* gene is thought to stimulate bone resorption and release calcium into the bloodstream.^(13)^ More recently, it has been suggested that FGF-23 and Klotho are not the only important factors in phosphate and vitamin Dhomeostasis.^(14)^ FGF-23 is a pleiotropic hormone that influences mineral metabolism and has recently been identified as an autocrine regulator of bone mineralization.^(8)^

This study primarily endeavored to assess whether Klotho and FGF-23 were associated with bone density in elderly individuals; nevertheless, no such correlation was observed in the study population.

Chalhoub et al.^(15)^ further investigated individuals belonging to the highest age group surveyed in this context and observed no association between α-Klotho and bone mineral density. Additionally, no association was noted between Klotho Score and bone loss or fracture incidence.

A previous Brazilian study conducted in the city of São Paulo, with the aim of evaluating *klotho* gene polymorphisms and correlating densitometric and fracture findings, did not reveal any substantial relationship between *klotho* gene polymorphisms and BMD or fractures.^(11)^

The absence of an association between α-Klotho and bone metabolism or bone mass could be enunciated by administering distinct dosages of different forms of the *klotho* gene. The Klotho membrane functions as an obligate coreceptor for FGF-23, which is reportedly involved in bone regulation. However, this type of Klotho cannot be readily measured.^(16)^

Koyama et al.^(10)^ attempted to delineate the relationship between Klotho and elderly individuals and observed that those with osteoporosis possessed lower levels of Klotho and, consequently, higher levels of FGF-23. Thus, these researchers concluded that aging, not CKD, is the principal regulatory factor of the *klotho* gene. Nevertheless, a different study failed to discover any association between FGF-23 levels and aging.^(17)^ In this study, FGF-23 demonstrated an association with CKD and strong correlations with poor bone quality and anemia in the elderly; however, there were no direct changes between FGF-23 and bone parameters.^(17)^

In the present study, no notable relationship was observed between Klotho levels and renal function. Among individuals undergoing hemodialysis owing to low kidney function, Klotho levels were lower in those with normal BMD than in those with low BMD (osteopenia).

The "Longevos Project"^(18)^ conducted with an identical cohort as the current investigation, demonstrated that FGF-23 was associated with age and renal filtration rate; nonetheless, this correlation was not verified in the present study. Moreover, Kužmová et al.^(19)^ documented that FGF-23 and Klotho were not associated with BMD but related to the trabecular bone score in the initial three stages of CKD; this indicated a probable association between the microarchitecture of the trabecular bone considerably earlier than with BMD. These findings may be elucidated by the limited number of CKD patients in the actual sample.

A significant constraint of this study is its limited sample size. Additionally, the scarcity of individuals with osteoporosis and normal BMD may have influenced the results, as the majority of the participants exhibited low BMD. Notwithstanding the restrictions, the present study solely included octogenarians (average age: 85 years), which should be highlighted as a strength. Alternatively, the absence of an association in the study population prompts the hypothesis that if the effects of Klotho vary over the lifetime of a person, the results presented may be validated irrespective of the impact of Klotho on aging.^(20)^

## CONCLUSION

No marked associations were observed between α-Klotho and FGF-23 levels and alterations in bone mineral density. Additional studies in an elderly population are warranted to evaluate the impact of α-Klotho and FGF-23 on bone health with aging.
